# Notices

**Published:** 1872-08

**Authors:** 


### *District Medical Society—Organization Completed.*

Pursuant to adjournment, the physicians of Central Illinois met in Farmer City, July 2nd, 1872, to complete the organization of "The Central Illinois Medical Society." Meeting called to order by Dr. C. T. Orner, chairman.

Reports of committees being the first thing in order, the committee on Constitution and By-Laws, through their chairman, Dr. Jno. Wright, submitted a copy, which, after one or two amendments, was unanimously adopted.

Committee on Nominations, through their chairman, Dr. Hill, reported:

For President—Dr. John Wright, Clinton.

For 1st Vice President—Dr. M. S. Brown, Urbana.

For 2nd Vice President—Dr. C. T. Orner, Saybrook.

For Corresponding Secretary—Dr. R. G. Laughlin, Bloomington.

For Recording Secretary—Dr. W. G. Cochran, Farmer City.

For Treasurer—Dr. H. C. Howard, Champaign.

For Censors—Dr. W. Hill, Bloomington; Dr. T. D. Fisher, LeRoy; Dr. J. T. Pearman, Champaign; Dr. C. Goodbrake, Clinton; Dr. J. S. Miller, Farmer City.

Report adopted.

Drs. Huddleston and Hill were appointed to conduct the President to his chair, when, by a few well-timed remarks, he thanked the society for the honor conferred upon him.

A motion that when we adjourn we adjourn to meet in Bloomington was carried unanimously.

Dr. R. G. Laughlin introduced the following resolution, which was adopted:

*Resolved*, That standing committees be appointed by the President on the subjects of Surgery, Practical Medicine, and Obstetrics, and that other standing committees be appointed at the option of the society.

In accordance therewith the following committees were appointed:

On Surgery—Dr. C. Goodbrake, Clinton; Dr. W. Hill, Bloomington; Dr. J. T. Pearman, Champaign.

On Practical Medicine—Dr. R. G. Laughlin, Bloomington; Dr. J. D. Gardner, Mahomet; Dr. T. K. Edmiston, Clinton.

On Obstetrics—Dr. S. H. Birney, Urbana; Dr. T. D. Fisher, LeRoy; Dr. J. S. Miller, Farmer City.

Committee of Arrangements—Drs. R. G. Laughlin, W. Hill, and R. D. Bradley, all of Bloomington.

Committee on Publication—Dr. W. G. Cochran, Farmer City; Dr. S. H. Birney, Urbana; Dr. W. Hill, Bloomington.

Dr. Laughlin offered the following resolution, which was unanimously adopted:

*Resolved*, That the thanks of the Central Illinois Medical Society are hereby tendered to the Good Templars for the gratuitous use of their hall, and to the profession and citizens of Farmer City for their cordial and generous hospitalities.

On motion of Dr. Hill, the Secretary was instructed to furnish copies of the proceedings for publication in the different county papers and the Chicago medical journals.

On motion, adjourned to meet in Bloomington, Illinois, on the second Tuesday in January, 1873. W. G. COCHRAN, *Sec'y.*

---

### *Circular—Illinois Institution for the Education of Feeble-Minded Children.*

This Institution, which was inaugurated in 1865 as an experimental school for the education of feeble-minded children, has been so successful in training this unfortunate class that at the last session of the General Assembly it was organized upon an independent basis, and was incorporated as one of the permanent charitable institutions of the State, thus completing the noble circle of public charities of the commonwealth of Illinois.

The design and object of the Institution is to furnish the means of education to children and youth of feeble minds, who are deprived of educational privileges elsewhere, and who are of a proper school-attending age. It is designed for those so deficient in intelligence as to be incapable of being educated at common schools, who are not epileptic, insane or deformed.

The education furnished by the Institution will include, not only the simpler elements of instruction usually taught in common schools, where that is practicable, but will embrace a course of training in the more practical matters of every-day life; the cultivation of habits of decency, propriety, self-reliance, and the development and enlargement of a capacity for useful occupation.

The combination which this Institution presents, of practical medical care and proper physical and mental training, with efficient educational resources, will supply, it is hoped, a want which has long been felt and imperatively demanded by this unfortunate class of children and youth of the State.

The improvement and progress of the pupils have been very encouraging, and parents and friends in almost every instance have expressed satisfaction with what has been accomplished in the short time since the school was organized.

The Institution is open to the inspection of the public at all reasonable hours; and all are not only cordially invited, but are earnestly requested to visit the school.

It is a State Institution, and board and tuition are free during the school year of ten months.

It is the desire of the Trustees to ascertain accurately the number of this unfortunate class of persons in the State, and persons knowing the residence of any in Illinois will confer a favor by reporting the same to the undersigned, as it is desirable that reliable statistics may be gathered in order that proper legislation may be made in their behalf.

The next school year will begin about the first of September, and those designing to apply for the admission of pupils should do so at once, as the accommodations are limited.

Applications for admission, information, etc., should be directed to

DR. C. T. WILBUR, *Superintendent,*
Jacksonville, Illinois.

